# Therapeutic Management of Advanced Hepatocellular Carcinoma: An Updated Review

**DOI:** 10.3390/cancers14102357

**Published:** 2022-05-10

**Authors:** Manon Falette Puisieux, Anna Pellat, Antoine Assaf, Claire Ginestet, Catherine Brezault, Marion Dhooge, Philippe Soyer, Romain Coriat

**Affiliations:** 1 Gastroenterology and Digestive Oncology Unit, Cochin Hospital, AP-HP Centre, 27 Rue du Faubourg Saint-Jacques, 75014 Paris, France; anna.pellat@aphp.fr (A.P.); antoine.assaf@aphp.fr (A.A.); claire.ginestet@aphp.fr (C.G.); catherine.brezault@aphp.fr (C.B.); marion.dhooge@aphp.fr (M.D.); romain.coriat@aphp.fr (R.C.); 2 Faculté de Médecine, Université Paris Cité, 75006 Paris, France; philippe.soyer@aphp.fr; 3 Radiology Department, Cochin Hospital, AP-HP Centre, 27 Rue du Faubourg Saint-Jacques, 75014 Paris, France

**Keywords:** hepatocellular carcinoma, cirrhosis, prognosis, treatment

## Abstract

**Simple Summary:**

Hepatocellular carcinoma is the fourth leading cause of cancer-related mortality worldwide and a major health problem. Overall survival is poor, with a five-year relative survival rate of 18.4% and only 2% in metastatic hepatocellular carcinoma. In 2020, the combination of atezolizumab and bevacizumab improved survival compared to sorafenib and was validated as the first-line treatment for advanced hepatocellular carcinoma. In case of disease progression, regorafenib and cabozantinib are recommended in the second-line setting. Transarterial chemoembolization can also be proposed for downstaging or in the palliative setting. Being able to reliably estimate liver function is a major issue in therapeutic management because patients with intermediate liver function are no longer eligible to receive systemic treatments. The aim of this review was to discuss systemic treatment management for patients with advanced unresectable HCC for whom liver-directed therapy is not appropriate.

**Abstract:**

Hepatocellular carcinoma (HCC) usually occurs in the setting of liver cirrhosis and more rarely in a healthy liver. Its incidence has increased in the past years, especially in western countries with the rising prevalence of non-alcoholic fatty liver disease. The prognosis of advanced HCC is low. In the first-line setting of advanced HCC, sorafenib, a tyrosine kinase inhibitor, was the only validated treatment for many years. In 2020, the combination of atezolizumab, an immune checkpoint inhibitor, and bevacizumab showed superiority to sorafenib alone in survival, making it the first-line recommended treatment. Regorafenib and lenvatinib, other multikinase inhibitors, were also validated in the second and first-line settings, respectively. Transarterial chemoembolization can be an alternative treatment for patients with intermediate-stage HCC and preserved liver function, including unresectable multinodular HCC without extrahepatic spread. The current challenge in advanced HCC lies in the selection of a patient for the optimal treatment, taking into account the underlying liver disease and liver function. Indeed, all trial patients present with a Child–Pugh score of A, and the optimal approach for other patients is still unclear. Furthermore, the combination of atezolizumab and bevacizumab should be considered in the absence of medical contraindication. Many trials testing immune checkpoint inhibitors in association with anti-angiogenic agents are ongoing, and primary results are promising. The landscape in advanced HCC management is undergoing profound change, and many challenges remain for optimal patient management in the years to come. This review aimed to provide an overview of current systemic treatment options for patients with advanced unresectable HCC who are not candidates for liver-directed therapy.

## 1. Introduction

Hepatocellular carcinoma (HCC) is the fourth leading cause of cancer-related mortality worldwide and a major health problem. HCC accounts for 80% of all cases of primary liver cancer, and its incidence increased to reach 660,000 new cases in 2018 worldwide, with 745,000 deaths. It is the fastest increasing cause of cancer-related death in the USA. The highest incidences are currently observed in Northern Africa, South-Eastern Asia, and Eastern Asia [1].

HCC usually occurs in the setting of cirrhosis, more rarely in non-cirrhotic chronic liver disease, and with the rare exception in a healthy liver. Hepatitis B virus is a major risk factor for HCC development and represents about 50% of cases of HCC. Non-alcoholic fatty liver disease (NAFLD) is actually the fastest increasing cause of cirrhosis in western countries [2]. When HCC occurs in the cirrhosis liver, the prognosis and the therapeutic approach depend on both tumor stage and liver function (Child–Pugh score).

Overall survival is poor, with a 5-year relative survival rate of 18.4%. The 5-year survival rates reach 33%, 10%, and 2% in patients with localized, regional, and metastatic disease, respectively [3].

The Barcelona Clinic Liver Cancer (BCLC) staging system uses different criteria to guide the therapeutic approach for patients with HCC. This score integrates the ECOG performance status, tumor burden (including portal invasion status and hepatic spread), and an evaluation of the underlying liver function that should be estimated beyond the Child–Pugh score using the Model for End-Stage Liver Disease score (MELD) in decompensated cirrhosis or alpha-fetoprotein (aFP) concentration and albumin-bilirubin score (ALBI) in compensated liver disease [4]. It allows us to divide patients with HCC in different disease stages into very early stage (BCLC 0), early stage (BCLC A), intermediate and unresectable stages (BCLC B and C), and end-stage (BCLC D, palliative care). At least 40% of HCCs are diagnosed at an early stage and are eligible for curative treatments, including surgical procedures (liver transplantation or hepatic resection) or local ablations with radiofrequency. However, more than half of the patients present intermediate-stage HCC (BCLC B and C), which requires chemoembolization, radioembolization, or systemic treatment.

In advanced HCC without vascular invasion (BCLC B), transarterial chemoembolization (TACE) should be considered for downstaging and can bring survival benefits [5,6]. However, there is a high recurrence rate following first-line treatment and repeated TACE may lead to liver dysfunction [7].

In earlier studies, systemic chemotherapy such as doxorubicin produced a response rate of about 10% but without any survival benefits [8]. Recent scientific advances have promoted the development of other systemic therapies, including tyrosine kinase inhibitors, monoclonal antibodies, and immune checkpoint inhibitors. For a long time, sorafenib, an oral multikinase inhibitor, was the only approved first-line treatment for advanced HCC with frequent dose reduction or discontinuation due to many adverse events [9,10]. Then, regorafenib became the second-line recommended treatment for patients treated with sorafenib [11]. Later, lenvatinib was considered non-inferior to sorafenib in a phase III trial [12]. However, all these therapeutics showed only modest improvement in survival [13,14].

In 2020, combined therapy with atezolizumab plus bevacizumab was shown to be superior to first-line sorafenib in unresectable HCC [15]. The natural history of advanced HCC involves a median survival of 8 months, and combination therapy more than doubled this life expectancy. It is now the first-line recommended treatment in advanced HCC.

Because of the emergence of both new therapeutic approaches and indications, it becomes difficult to rely solely on the BCLC staging system. In the short term, new treatment perspectives will deeply challenge our current therapeutic approach. The aim of this review is to discuss systemic treatment management for patients with advanced unresectable HCC (BCLC B or C) for whom liver-directed therapy is not appropriate.

## 2. Tyrosine Kinase Inhibitors in Advanced Hepatocellular Carcinoma

**Figure 1:**
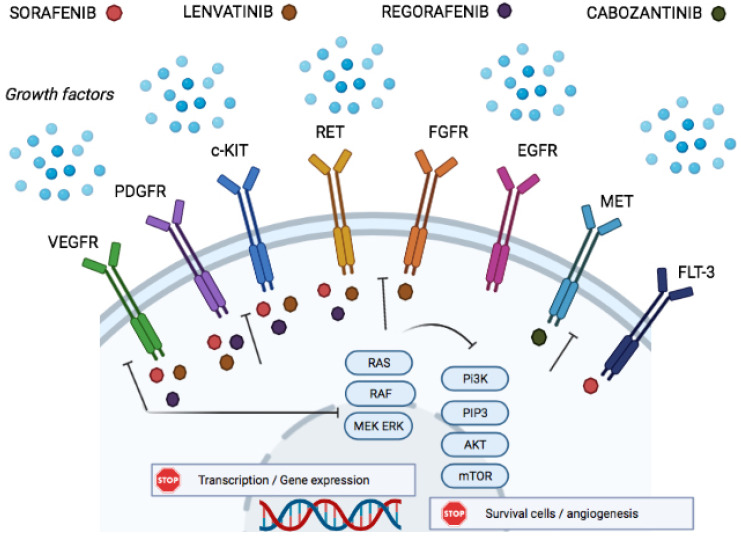
Tyrosine kinase inhibitors and molecular pathways in hepatocellular carcinoma.

Angiogenesis is a determining factor for tumor growth, and several angiogenic pathways are involved in the development of liver cancer [16]. In particular, the vascular endothelial growth factor receptor (VEGFR) is overexpressed in HCC and leads to an abnormal conformation of tumoral blood vessels, causing abnormal blood flow and a lack of oxygen delivery. This overexpression of VEGFR is associated with poor clinical outcomes, suggesting that it could be involved in liver cancer pathogenesis and could be a therapeutic target [17]. Tyrosine kinases are involved in the activation of a wide range of protein phosphorylations. Tyrosine kinases inhibitors (TKI) block the active sites, thus preventing phosphorylation and inhibiting the downstream signal transduction of a range of growth factors, such as VEGFR and epidermal growth factor receptor (EGFR 2). This induces an increase in the rate of tumor apoptosis (Figure 1).

In 2007, sorafenib was the first oral multikinase inhibitor to receive approval for systemic treatment of advanced HCC (BCLC B and C). This drug inhibits the activity of vascular endothelial growth factor receptors (VEGFR2, VEGFR3) and platelet-derived growth factor receptors (PDGFR). In 2008, a phase III trial (SHARP) enrolled patients with advanced HCC who had not received previous systemic treatment and presented Child–Pugh A liver function. A total of 602 patients were randomly assigned to receive either sorafenib 400 mg twice daily or a placebo. Overall survival was higher in the sorafenib group, 10.7 months versus 7.9 months in the placebo group (HR 0.69, 95% CI 0.55 to 0.87, *p* < 0.001) [9]. No significant difference was shown in the median time to progression between the two groups (4.1 months versus 4.9 months, HR 1.08, 95% CI 0.88 to 1.31, *p* = 0.77), but a significant difference in radiologic progression was shown (5.5 months versus 2.8 months, *p* < 0.001). The main adverse events in the SHARP trial were hand-foot syndrome (7.0%), asthenia (7.4%), and diarrhea (13.1%), which can have a significant impact on quality of life and may lead to a decrease or cessation of the drug in 26% and 44% of cases, respectively. These results were subsequently confirmed in a randomized controlled trial in the Asia Pacific in 2009. The median overall survival was 6.5 months versus 4.2 months in the placebo group (HR 0.68, 95% CI 0.50 to 0.93, *p* = 0.014). The median time to progression was also improved: 2.8 months versus 1.4 months (HR 0.57, *p* = 0.005) [18].

For ten years, sorafenib was the only first-line systemic treatment shown to provide an improvement in overall survival in advanced HCC for patients with Child–Pugh A liver function. All phase III trials testing new systemic drugs had failed to improve survival in the first-line (sunitinib, linifanib, or doxorubicin) [19,20,21] or second-line settings (Brivanib or ADI peg 20) [22,23]. Many reasons can explain these failures, but they are largely related to inadequate consideration of the underlying liver disease. As an example, sunitinib had major liver toxicity at the tested posology (37.5 mg once per day). The underlying etiology of the liver disease (alcoholic, non-alcoholic fatty liver disease called NAFLD, viral hepatitis), vascular invasion, or extrahepatic metastasis were all underestimated factors which led to treatment failure in these trials.

The RESORCE trial evaluated regorafenib in patients with HCC who progressed on sorafenib. Regorafenib is another oral multikinase inhibitor (VEGFR1-3, c-KIT, PDGFR, FGFR, RET, RAF, BRAF, and p38 MAP kinase) that is approved in refractory metastatic colorectal cancer and gastrointestinal stromal tumors (GISTs). This randomized, double-blind, placebo-controlled phase 3 trial showed a benefit in terms of overall survival in patients with preserved liver function (Child–Pugh A): 10.6 months versus 7.8 months for placebo (HR 0.63, 95% CI 0.50 to 0.79, *p* < 0.0001) [11]. Regorafenib was prescribed 3 out of 4 weeks at a dose of 160 mg once daily. Following these results, regorafenib was recommended as second-line systemic therapy after progression on sorafenib. Regarding adverse effects, hand-foot skin reaction with regorafenib seems to be associated with improved overall survival, as it was previously shown for sorafenib (14.1 months versus 6.6 months when no hand-foot skin reaction, HR 0.52, 95% CI, 0.40 to 0.67) [24].

Considering the lack of systemic treatment options for patients with advanced HCC, lenvatinib was also evaluated in the first-line setting for the treatment of advanced HCC. Lenvatinib is another oral TKI that targets VEGF, FGF, PDGF, RET, and KIT receptors and has shown to be effective in advanced renal cell carcinoma and differentiated thyroid cancer. In a phase II study in HCC, 12 mg lenvatinib once daily showed clinical activity and had an acceptable safety profile [25]. In 2018, the phase III REFLECT trial displayed that lenvatinib was not inferior to sorafenib in overall survival. Indeed, the median survival time for lenvatinib (8 mg for bodyweight < 60 kg or 12 mg once daily) of 13.6 months was non-inferior to sorafenib (12.3 months, HR 0.92, 95% CI 0.79 to 1.06), meeting criteria for non-inferiority [12]. The median time to progression was 8.9 months compared to 3.7 months in the sorafenib group, and an improvement was also shown in terms of progression-free survival and objective response rate according to the mRECIST score. The arterial hypertension rate was higher in the lenvatinib group (23% versus 14% in the sorafenib group), but fewer hand-foot skin reactions (3% versus 11%) were observed. With these results, lenvatinib was approved as an alternative first-line agent in unresectable HCC. For example, in case of severe hand-foot skin reaction with sorafenib, a switch to lenvatinib should be considered, and lenvatinib should be preferred in patients with many cardiovascular comorbidities due to the risk of severe arterial hypertension and ischemic strokes [26].

In cells exposed to sorafenib, an over-expression of c-MET has been described and could be an explanation for disease progression. Cabozantinib, a VEGFR and c-MET inhibitor, was tested in the CELESTIAL phase III trial that included 707 patients with HCC who progressed on sorafenib. Patients were randomized to receive either cabozantinib 60 mg once daily or a placebo. Overall survival with cabozantinib was 10.2 months and 8.0 months with placebo (HR 0.76, 95% CI 0.63 to 0.92, *p* = 0.005). Progression-free survival was also significantly improved in the cabozantinib group (5.2 months versus 1.9 months, HR 0.44; 95% CI 0.36 to 0.52, *p* < 0.001) [27]. The most common adverse effects leading to cessation or dose reductions of cabozantinib were hand-foot skin reaction (22%), diarrhea (10%), asthenia (7%), and arterial hypertension (7%). Considering these results, cabozantinib should be considered in patients with sorafenib intolerance or who progressed after previous systemic treatments.

## 3. Monoclonal Antibodies in Advanced Hepatocellular Carcinoma

**Figure 2:**
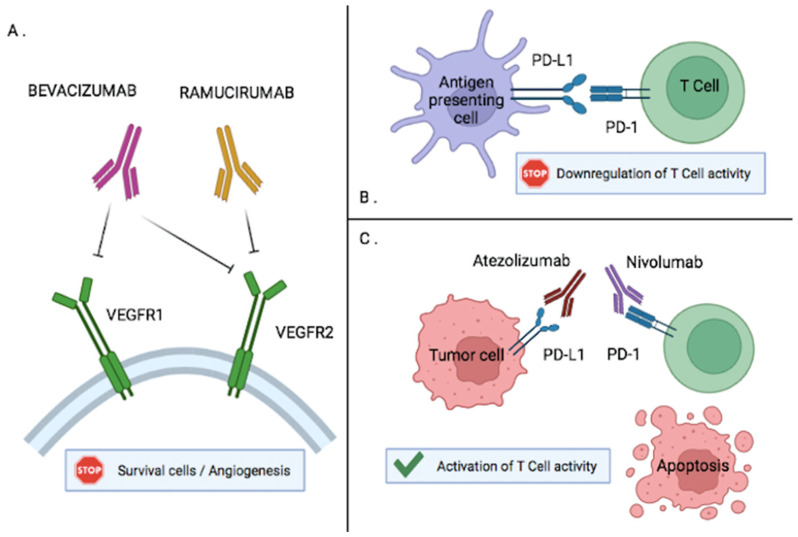
(**A**). Anti-VEGF and monoclonal antibody (ramucirumab) inhibit angiogenesis in tumor cell (**B**). Activated T-cells express P-1 that binds to its specific ligand PD-L1 on antigen presenting cell which inhibits T-cell activity (**C**). Anti PD-1 (Nivolumab) and anti-PD-L1 (Atezolizumab) prevent this binding and increase T-cell activation which allows apoptosis of tumor cells.

Ramucirumab is a human recombinant IgG1 monoclonal antibody that inhibits receptor activation of VEGFR2 (Figure 2A). In the REACH trial, 565 patients with Child–Pugh A liver function and presenting an intolerance or progression on sorafenib were randomized to receive ramucirumab 8 mg/kg intravenously every 2 weeks or a placebo. Overall survival with ramucirumab was 9.2 months versus 7.6 months in the placebo group (HR 0.87, 95% CI, 0.72 to 1.05, *p* = 0.14) [28]. Even if progression-free survival was improved, these results were non-inferential because no significant difference in overall survival was shown. In subgroup analysis, overall survival seems to be improved in patients with a baseline alpha-fetoprotein (aFP) concentration of 400 ng/mL or greater (7.8 months versus 4.2 months in the placebo group), as well as median progression-free survival (2.7 months versus 1.5 months in the placebo group). It is well known that an aFP concentration higher than 400 ng/mL is associated with a poor prognosis in patients with advanced HCC [29,30]. These results suggested an increasing effect of ramucirumab with an increasing value of aFP.

This is why the REACH-2 trial randomized patients with an aFP baseline > 400 ng/mL and Child–Pugh A liver function to receive either ramucirumab or a placebo. Overall survival was significantly improved in the ramucirumab group (8.5 months versus 7.3 months, HR 0.710, 95% CI 0.531 to 0.949, *p* = 0.0199) [31]. The most common adverse events in the ramucirumab group were bleeding (3.5%) and arterial hypertension (12%).

A pooled analysis of results from both REACH and REACH 2 trials showed benefits in terms of disease-related symptoms and health-related quality of life. Time to deterioration was improved in the ramucirumab group (3.3 months versus 1.9 months in the placebo group, HR 0.725, 95% CI 0.559 to 0.941, *p* = 0.0152), and patients presented less back pain, weight loss, and pain [32]. Ramucirumab is an alternative treatment in second-line therapy for advanced HCC, but the Marketing Authorization Approval extension has not been approved in Europe.

Bevacizumab is an anti-VEGF agent that inhibits tumor growth. A phase II study showed response rates of about 13 to 14% with bevacizumab in patients with advanced HCC [33] (Figure 2A).

## 4. Immune Checkpoint Inhibitors in Advanced Hepatocellular Carcinoma Management

Anti-VEGF therapies bind to specific receptors (VEGFR1 or VEGFR2) and inhibit angiogenesis in tumor cells. Antigen-presenting cells express PD-L1 and bind to its ligand (PD-1) on T-Cells. This binding is responsible for the downregulation of T-cell activity (Figure 2B,C). Systemic immune checkpoint inhibitors, such as atezolizumab (anti-PD-L1) or nivolumab (anti-PD-1), prevent this binding and increase T-cell activation to restore antitumoral activity [34,35,36]. Various immune checkpoint inhibitors have been evaluated for the treatment of advanced HCC.

Nivolumab is an immunoglobulin-g4 monoclonal antibody anti-PD-1 immune checkpoint. Based on the phase I—II CheckMate 040 trial, nivolumab, given at the dose of 3 mg/kg, brings an objective response rate of about 20% for patients with advanced HCC and Child–Pugh A liver function after sorafenib failure [37]. In this trial, the disease control rate (DCR) was 64%, and progression-free survival was 4.1 months with nivolumab. Recently, the phase III CheckMate 459 trial randomized 743 patients to receive either nivolumab (240 mg intravenously every 2 weeks) or sorafenib (400 mg orally twice daily). Median overall survival was not improved in the nivolumab group: 16.4 months versus 14.7 months with sorafenib (HR 0.85, 95% CI, 0.72 to 1.2, *p* = 0.075) [14]. The safety profile was satisfying, and the most common adverse effects were hand-foot skin reaction (1%) and aspartate amino acid transferase increase (6%). Nivolumab was also compared to regorafenib as a second-line systemic treatment in patients with advanced HCC and sorafenib failure [38]. No significant difference was observed between the two groups in terms of overall survival, time to progression, or objective response rate.

Pembrolizumab, another anti-PD-1 monoclonal antibody, was also evaluated in patients with advanced HCC. In 2019, the phase III KEYNOTE-240 trial enrolled 413 patients with HCC with progression or intolerance to sorafenib. Patients were randomly assigned to receive either pembrolizumab (200 mg every 3 weeks) or a placebo. Objective response rate was 18.3% in the pembrolizumab group versus 6% with the placebo (*p* = 0.00007). The median overall survival was 13.9 months for pembrolizumab and 10.6 months for placebo (HR 0.78, 95% CI, 0.611 to 0.998, *p* = 0.0238). Progression-free survival was 3.0 months and 2.8 months with pembrolizumab and the placebo, respectively (HR 0.71, 95% CI, 0.570 to 0.904, *p* = 0.0022). Although overall and progression-free survivals both improved with pembrolizumab compared with the placebo, statistical significance was not reached (*p* = 0.0174 for overall survival and *p* = 0.002 for progression-free survival), and these results are consistent with those of the phase II KEYNOTE-224 [39]. Pembrolizumab is not approved in second-line treatment for advanced HCC.

Atezolizumab targets PD-L1 to prevent interaction with PD-1 and B7-1 receptors. In a multicenter, open-label, phase III randomized trial conducted in 2020 (IMbrave150), 501 patients were randomized to receive either 1200 mg of atezolizumab plus 15 mg/kg of bevacizumab intravenously every 3 weeks or sorafenib (400 mg orally twice daily) [15]. Overall survival at 6 months and median progression-free survival were both improved: 84.8% versus 72.2% and 6.8 months versus 4.3 months (HR 0.59, 95% CI, 0.47 to 0.76, *p* < 0.001) in the atezolizumab-bevacizumab group and sorafenib group, respectively. Stratified hazard ratio for death with atezolizumab-bevacizumab as compared with sorafenib was 0.58 (95% CI 0.42 to 0.79, *p* < 0.001). Finally, objective response rates according to RECIST were 27.3% and 11.9% in the atezolizumab-bevacizumab and sorafenib groups, respectively. The most common adverse event was hypertension (15.2%). To be noted, some cases of esophageal varices hemorrhage (7% versus 4.5% in the sorafenib group), gastrointestinal disorders, and interstitial pneumonia were reported. Because upper gastrointestinal bleeding is a life-threatening complication, the presence of gastric or esophageal varices has to be evaluated before the prescription of this treatment. Following these results, the combination therapy of atezolizumab with bevacizumab is now validated as a first-line treatment in advanced HCC.

**Figure 3:**
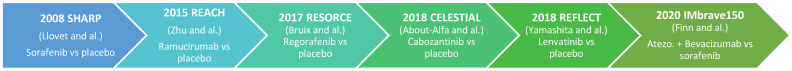
Historical timeline of systemic therapies approved in advanced hepatocellular carcinoma management [9,11,12,15,27,28].

**Figure 4:**
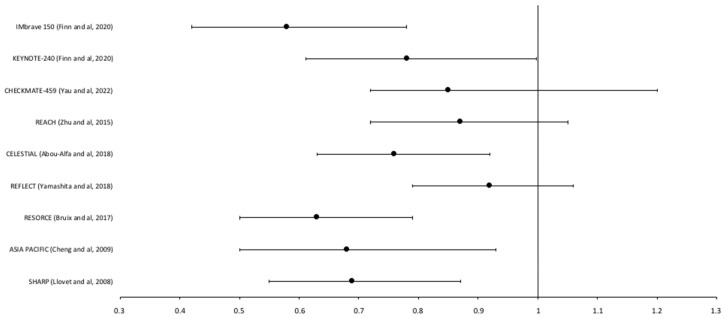
Forest plots of hazard ratio (HR) of overall survival (OS) in phase III-studies on advanced HCC [9,11,12,13,14,15,18,27,28].

A summary of the different systemic therapies evaluated in advanced HCC over time is represented in Figure 3 and Figure 4.

## 5. Perspectives of New Combination Therapies

Promising results have been shown when targeted therapies are associated with anti-angiogenic agents. A phase Ib trial evaluated the combination of lenvatinib and pembrolizumab and showed promising results with improved progression-free survival of 9.5 months and overall survival of 22 months [40]. A phase III study comparing lenvatinib plus pembrolizumab to lenvatinib plus placebo is in progress (NCT03713593).

The COSMIC-312 phase III trial tested the combination therapy of atezolizumab and cabozantinib compared to sorafenib alone. A total of 837 patients with Child–Pugh A liver function and advanced HCC were enrolled. Median progression-free survival was significantly improved at 6.8 months with combination therapy versus 4.2 months with sorafenib (HR 0.63, *p* = 0.0012). The risk of disease progression also decreased by about 37%. However, no significant differences were observed for the primary overall survival endpoint in the intention-to-treat population (15.4 months with combination therapy vs. 15.5 months with sorafenib, HR 0.90, *p* = 0.438) [41].

Furthermore, CTLA4 inhibition in combination with PD-1 or PD-L1 inhibition could be an interesting way to improve the response rate duration. A HIMALAYA phase III trial (NCT03298451) is in progress to compare durvalumab (anti-PD-L1) with or without tremelimumab (anti-CTLA4) to sorafenib in first-line treatment of advanced HCC. Primary results showed that overall survival was significantly improved in the durvalumab plus tremelimumab group compared to the sorafenib group (16.4 months versus 13.8 months HR 0.78, 96% CI, 0.65–0.92, *p* = 0.0035). This immunotherapy combination also displayed favorable safety (25.8% grade 3–4 adverse events versus 36.9% in the sorafenib group) and could become an alternative first-line agent.

A phase III trial is also ongoing for the evaluation of nivolumab and ipilimumab as first-line therapy.

## 6. Interventional Radiology

According to the BCLC staging system, TACE is the recommended treatment for patients with intermediate-stage HCC (without vascular invasion or metastasis) and preserved liver function (Child–Pugh A, BCLC B), including unresectable multinodular HCC without extrahepatic spread [42].

Even if TACE can be used in combination with curative treatments (while waiting for a liver transplant or to obtain a downstaging), it has been validated as palliative care following the results of a meta-analysis showing an improvement of 2-year survival compared with control (OR 0.53, 95% CI, 0.32 to 0.89, *p* = 0.017) [43]. Improvements in the selection of patients and the use of selective embolization to reduce collateral hepatic toxicity have led to current median survival times of 26 months and beyond 40 months [44,45] in patients with unresectable HCC. However, the high recurrence rate after TACE is a major limitation, possibly resulting from increased expression of VEGF [46]. Less than 20% of patients initially treated by TACE will be able to receive systemic treatment because repetitive TACE can lead to a deterioration of liver function, making patients ineligible for future drug-based treatments [47,48].

Choosing between systemic therapeutics and TACE is a source of discussion. According to ESMO guidelines, systemic treatment should be preferred in patients with large tumors (>10 cm), multinodular HCC (7–8 tumors), bilobar HCC, and an elevated aFP score [49].

As sorafenib reduces angiogenesis and proliferation of tumor cells, combining sorafenib and TACE was a promising strategy. This combination was evaluated in several trials, and a meta-analysis of 27 phase I and II trials showed that DCR and time to progression in the combination therapy group were significantly improved compared to TACE alone (OR 2.93, 95% CI 1.59 to 5.41, *p* = 0.005 and HR 0.66, 95% CI 0.50 to 0.81, *p* = 0.002, respectively). However, combination therapy did not significantly improve overall survival (HR 0.63, 95% CI 0.55 to 0.71, *p* = 0.058) [46].

A phase III, multicenter, randomized trial (LAUNCH) evaluated a combination of lenvatinib and TACE versus lenvatinib alone as first-line treatment in patients with advanced HCC and Child–Pugh A liver function. Inclusion criteria were a single lesion sized < 10 cm or less than 10 lesions. A total of 170 patients were enrolled in the lenvatinib plus TACE group and 168 patients in the lenvatinib alone group. Primary results showed that the overall response rate and disease control rate were significantly higher in the lenvatinib plus TACE group. Overall survival also seemed to improve in the combination therapy group with 17.8 months versus 11.5 months, respectively (HR 0.45, 95% CI 0.33 to 0.61, *p* < 0.001). After discontinuation, curative surgical treatment was conducted in 26 patients in the combination group, owing to downstaging, versus 3 patients in the lenvatinib only group (*p* < 0.001). Lenvatinib plus TACE may be a potential new first-line treatment option for advanced HCC.

Selective internal radiation therapy (SIRT) with yttrium-90 microspheres, also called radioembolization, delivers therapy through the hepatic artery to liver tumors. In 2009, a study retrospectively showed that median survival was significantly improved for patients who received radioembolization compared to the control (16 months versus 8 months, *p* < 0.05) [50]. Survival varied significantly by performance status scale, liver function (Child-Pugh), number of tumors, and presence of extrahepatic disease [51,52,53]. The SARAH multicenter, randomized trial compared sorafenib to internal radiation therapy with yttrium-90 microspheres in patients with unresectable HCC. Overall survival did not significantly differ between the two groups (8 months in the SIRT group, vs. 9.9 months in the sorafenib group, HR 1.15, 95% CI 0.94 to 1.41, *p* = 0.18) [54]. Even if adverse events were less common in the radioembolization group, particularly diarrhea and asthenia, the lack of benefits on overall survival did not allow for radioembolization to be considered an alternative first-line treatment for advanced HCC. The SIRveNIB trial conducted in Asia-Pacific patients was also negative [55]. Similarly, in the SORAMIC trial, which randomly assigned 216 patients to SIRT plus sorafenib and 208 patients to sorafenib alone, overall survival did not differ (HR 1.01, 95% CI 0.81 to 1.25, *p* = 0.9529) [56]. As all these randomized phase III trials reported negative results, a phase II trial aimed to compare the efficacy of personalized dosimetry (≥205 Gy targeted to the index lesion) versus the standard dosimetry approach of selective internal radiation therapy. Patients were eligible if they had an unresectable advanced HCC, at least one measurable lesion of 7 cm or more in size, and no extrahepatic spread. A dose of radiation ≥ 205 Gy improved response rate, the possibility of secondary resection, and overall survival (26.6 months versus 10.7 months, *p* = 0.0096) [57]. A secondary retrospective analysis of prospectively acquired data from participants of the SARAH trial was performed to determine the relationship between tumor radiation-absorbed dose and survival and tumor response. A higher tumor radiation-absorbed dose was associated with better overall survival and disease control [58].

Radioembolization is actually recommended for patients with BCLC B or C HCC who are not eligible for sorafenib or after progression with sorafenib with portal invasion but preserved liver function (Child–Pugh A).

**Figure 5:**
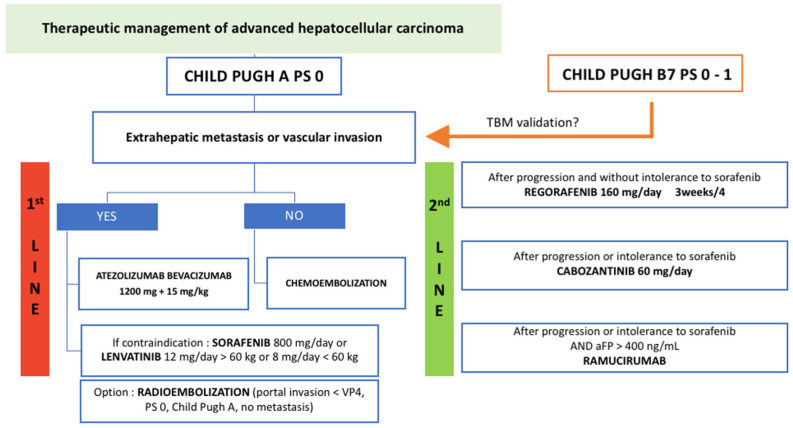
Algorithm proposition for therapeutic management of advanced hepatocellular carcinoma in patients with Child–Pugh A liver function. TBM: tumor board meeting.

An algorithm for therapeutic management of advanced hepatocellular carcinoma is proposed in Figure 5.

## 7. What Is the Status of Surgical Treatment in Metastasis of Hepatocellular Carcinoma?

The 5-year survival rate of patients with metastatic HCC is poor, about 2%. The most frequent sites of extrahepatic metastases are the lungs, followed by lymph nodes, bones, and the adrenal glands. Average survival is about 3 months once a pulmonary metastasis is detected [59]. While the resection of isolated metastasis brings a survival benefit for certain malignancies, the role of extrahepatic metastasectomy in HCC is not well-established. A retrospective study enrolled 85 patients who underwent extrahepatic metastasectomy procedures (lung resections, peritoneal cytoreductive surgeries, lymphadenectomies, adrenalectomies, bone resections), the majority following liver resection or transplantation [60]. The median overall survival was 25.3 months, and the median progression-free survival was 7.7 months. Of note, patients who underwent lung metastasectomy had better overall survival compared with other extrahepatic metastasectomy sites (36.6 months versus 23.6 months, *p* = 0.039). Overall survival was also improved in patients who underwent metastasis resections compared to patients treated with sorafenib alone (27.7 versus 7.4 months, *p* < 0.001). A Japanese study included patients that were good candidates for resection of pulmonary metastases from HCC: possibility of complete resection, no evidence of uncontrolled intrahepatic or extrapulmonary lesions, and adequate general condition [61]. A total of 39 out of 45 patients underwent hepatectomy or liver transplantation, and 6 underwent locoregional therapy for primary liver tumors. In total, 21 patients had a history of intra or extrahepatic recurrence before initial pulmonary metastasectomy, and 24 patients did not. Overall survival was 26.5 months, and 2- and 5-year overall survival rates were 53.9% and 40.9%, respectively.

Therefore, it is important to identify clinical parameters to better select patients who will be good candidates for metastasectomy. The presence of more than two extrahepatic lesions, a Child–Pugh score > A5, aFP level > 100 ng/mL, metastatic site other than the lung, and a size > 3 cm for the largest resected metastasis seem to be unfavorable prognostic factors and associated with poor survival.

Remission status in the liver before pulmonary metastasis resection and a distant-metastasis-free interval of >6 months before metastasectomy are probably important factors associated with improved overall survival [59].

Further studies are still needed to identify patients who may really benefit from metastasectomy for lung metastasis of HCC. Currently, the decision is based on a discussion in Tumor Board Meetings on a case-by-case basis in patients with controlled liver disease.

## 8. Management of the Underlying Liver Disease

HCC usually develops from chronic liver disease, and it is well known that the overall survival of patients is influenced by tumor stage (BCLC score), underlying-liver function (Child–Pugh score), and ECOG performance status. A Child–Pugh score > A is an exclusion criterion for all phase III trials in advanced HCC, and recommendations are not clear for patients with intermediate liver function.

It is therefore essential to control the underlying liver disease and optimize the management of cirrhosis complications such as portal hypertension and ascites.

Etiological treatment of liver disease is a key point before starting systemic treatment for HCC. Alcohol consumption should be stopped, cardiovascular risk factors should be controlled, and virus B hepatitis should be treated. Initially, the benefit of eradicating the hepatitis C virus was uncertain in patients with advanced HCC [62], but a recent retrospective cohort of 168 patients who received sorafenib showed that a survival benefit was probably conferred by HCV eradication [63].

Being able to reliably estimate liver function is also a major issue in the therapeutic management of HCC because patients with a Child–Pugh score > B7 are no longer eligible to receive systemic treatments and will only receive palliative care. However, the Child–Pugh score may be difficult to estimate in patients with advanced HCC because the degree of liver dysfunction could be related to both the tumor stage and the underlying-liver condition. Moreover, degrees of ascites and encephalopathy are subjective criteria and discrimination between mild and moderate can be slight.

The albumin-bilirubin (ALBI) model was proposed in 2015 to better assess liver function in these patients and highlight prognostic subgroups in Child–Pugh A patients [64]. To identify objective measures and combine them for this model, data from major HCC centers in Japan, the United Kingdom, Spain, China, and the United States and from international HCC trials were reported. The study showed that vascular invasion, albumin, tumor size, log10 bilirubin, tumor number, age, and sex were significant prognostic variables. When the impact of HCC itself was excluded, only albumin and bilirubin remained significant predictors of survival. The ALBI model was cut into 3 different grades: x ≤ −2.60 (ALBI grade 1), −2.60 to ≤−1.39 (ALBI grade 2), and x > −1.39 (ALBI grade 3).

When applying this model to all of the cohorts, overall survival curves were similar to those estimated with the Child–Pugh score. In contrast, for patients with Child–Pugh A liver function who received sorafenib, the model could discriminate between a favorable risk group (ALBI grade 1) and an unfavorable risk group (ALBI grade 2). There was a median survival difference of nearly 6 months between these two ALBI grades. Recently, a phase II study compared sorafenib to palliative care in patients with a Child–Pugh B liver function. No significant difference in overall survival was shown for Child–Pugh B patients who received sorafenib. However, patients with ALBI 1 or 2 grades could benefit from sorafenib [65]. Overall, sorafenib could be discussed on a case-by-case basis for patients with B7 Child–Pugh Score.

## 9. Conclusions

In advanced HCC management, new therapeutics have emerged over the past decade. Based on the results of phase III trials, six systemic therapies have been approved as first or second-line treatment. The combination of atezolizumab and bevacizumab should be preferred in the first-line setting of advanced HCC in the absence of medical contraindication and in patients with Child–Pugh A liver function. The time to deterioration in quality of life is improved (11.2 months), but survival benefit remains poor (progression-free survival 6.8 months, overall survival 67.2% at 12 months). The therapeutic approach for patients with a B7 Child–Pugh score should be individually discussed in specialized Tumor Board Meetings. Many trials testing immune checkpoint inhibitors in association with anti-angiogenic agents are ongoing, and primary results are promising. The landscape in advanced HCC management is undergoing profound change, and many challenges remain for optimal patient management in the years to come.
